# Quality Index for Stereoscopic Images by Separately Evaluating Adding and Subtracting

**DOI:** 10.1371/journal.pone.0145800

**Published:** 2015-12-30

**Authors:** Jiachen Yang, Yancong Lin, Zhiqun Gao, Zhihan Lv, Wei Wei, Houbing Song

**Affiliations:** 1 School of Electronic Information Engineering, Tianjin University, 92 Weijin Road, Tianjin, 300072 China; 2 Shenzhen Institute of Advanced Technology, Chinese Academy of Sciences, 1068 Xueyuan Avenue, Shenzhen University Town, Shenzhen, 518055 China; 3 School of Computer Science and Engineering, Xi’an University of Technology, Xi’an, Shaanxi 710048 China; 4 Department of Electrical and Computer Engineering, West Virginia University, Montgomery, WV 25136 United States of America; Beijing University of Technology, CHINA

## Abstract

The human visual system (HVS) plays an important role in stereo image quality perception. Therefore, it has aroused many people’s interest in how to take advantage of the knowledge of the visual perception in image quality assessment models. This paper proposes a full-reference metric for quality assessment of stereoscopic images based on the binocular difference channel and binocular summation channel. For a stereo pair, the binocular summation map and binocular difference map are computed first by adding and subtracting the left image and right image. Then the binocular summation is decoupled into two parts, namely additive impairments and detail losses. The quality of binocular summation is obtained as the adaptive combination of the quality of detail losses and additive impairments. The quality of binocular summation is computed by using the Contrast Sensitivity Function (CSF) and weighted multi-scale (MS-SSIM). Finally, the quality of binocular summation and binocular difference is integrated into an overall quality index. The experimental results indicate that compared with existing metrics, the proposed metric is highly consistent with the subjective quality assessment and is a robust measure. The result have also indirectly proved hypothesis of the existence of binocular summation and binocular difference channels.

## Introduction

In the past several years, we have witnessed a rapid development of stereo and multi-view system and a widespread application of these systems [1–4]. However, various distortions may be introduced during the creation, transmission, processing and display of stereoscopic contents, which will affect the quality perception and result in the visual fatigue and visual discomfort [5, 6]. Consequently, it is important to build an effective tool to measure the quality of stereoscopic images. Stereoscopic image quality assessment (SIQA), which is similar to the two dimensional (2D) quality assessment (2D-IQA), can be categorized into subjective and objective methods [7]. Since human eyes are the terminal receiver of visual signals, it is reasonable to regard the subjective assessment, which represents the directional reflection of the HVS, as the most convincing way to evaluate image quality. During the recent year, the research on the subjective experiment has processed steadily, and various factors that may affect the stereoscopic perception have been found and investigated. Zhou offered a standard procedure to evaluate the distortions introduced during the process of transmission and compression [8]. Lee *et al*. proposed a paired comparison based on the subjective experiment which intended to minimize the effect of the subject’s limited 3D experience [9]. IJsselsteijn *et al*. investigated how the camera configurations settings impact the visual quality [10]. Wöpking *et al*. figured out the threshold of the binocular disparity and the depth of focus that may cause viewing discomfort [11]. However, the subjective tests based on the specific standard procedure need repeated experiments with a great number of participants [12]. Therefore, the definition of the objective metric, which can be used to reliably predict the perceived quality of stereo images, has drawn great attention of scholars and experts.

However, designing an accurate objective metric is not an easy matter. There are many challenges. Firstly, during the procedure of image processing, various distortions are introduced, if not impossible. It is hard to design a metric that is effective for every type of distortion. Secondly, since the visual signals have various contents, it will lead to sophisticated distortion masking. Lastly, after entering the eyes, the visual signals are decoupled and interpreted by the HVS. However, the HVS and its mathematical modeling have not been realized due to the great complexity. Nevertheless, the research on SIQA has aroused many experts’ efforts to make it a reality.

The 2D-IQA has been widely researched and there are many quality assessment metrics such as structural similarity (SSIM) [13], peak signal-to-noise ratio (PSNR) and visual information fidelity (VIF) [14]. A straightforward way is to extend these 2D metrics to SIQA. The earliest pioneers who attempted to evaluate the quality of stereo images followed this strategy. Boev *et al*. combined the monoscopic quality component and the stereoscopic quality component for stereo quality assessment [15]. Considering that the stereoscopic images are different from the plane images because of the additional information of depth, YOU *et al*. investigated the capabilities of some common 2D quality metrics in SIQA, and added the depth information into quality assessment [16]. A similar approach was proposed by Benoit *et al*., where two measures of 2D quality SSIM and C4 were augmented with disparity information by computing the square root of the difference between the reference and distorted disparities to predict the quality. However, these metrics are inefficient in predicting the perceived quality because the stimuli toward the perceived depth are quite different from those for 2D image quality [17]. Bosc *et al*. first questioned if it was appropriate to use 2D objective metrics and 2D subjective protocols to evaluate the quality of 3D contents [18].

During the recent years, the concept of Quality of Experience was proposed and widely used to reflect the overall quality experience of the subjects that were accessing and using the service provided. Some experts introduced this concept in the SIQA. The QoE of 3D refers to the unique aspects such as visual comfort, visual fatigue and other visual experience. Chen *et al*. proposed a 3D QoE by constructing visual experience as a weight sum of the image quality, depth quality and visual comfort [19]. Lin et al. adopted three quality components in their metric to evaluate the quality of stereoscopic video, namely the cyclopean view, binocular rivalry, and the scene geometry [20]. Qi used the relationship between the left and right views to reflect the QoE, and represented the structure of low-level features by using the phase congruency and saliency map [21].

Furthermore, some authors have taken a perceptual route in the development of SIQA algorithms. Shen *et al*. transformed the image by using the Laplacian pyramid and a directional filter-bank to simulate the multi-channels of HVS and then computed the Minkowski summation of the error between the reference and distorted images to derive a quality [22]. Maalouf *et al*. integrated the left and right images into a cyclopean image to simulate the brain perception, and used the contrast sensitivity coefficients of the cyclopean image to lead a quality [23]. Ha et al. proposed a scheme for quality assessment of stereo images by considering the factors of temporal variation and disparity distribution [24]. Wang *et al*. proposed a SIQA metric by taking into account of the binocular spatial sensitivity to reflect the binocular fusion and suppression properties [25]. Bensalma *et al*. modeled the simple cells and complex cells of vision system, where the quality is given as the difference of associated binocular energy [26]. Shao *et al*. divided stereo images into monocular region, binocular fusion region and binocular suppression region, where the overall quality is given as the linear summation of three regions [27]. All the above metrics considered the characteristics of the HVS, either low-level or high-level. However, the key step of those metrics considering low-level characteristics of the HVS is stereo matching which needs an enormous quantity of calculations and is still an unsolved problem in stereo-related research. High-level HVS processing mechanism is still mysterious. Theories used in these models are generally the common knowledge and assumptions about the high-level HVS characteristics, which makes their quality prediction not convincing.

Recently, a new study has found the evidence that the HVS has separately adaptable channels for adding and subtracting the neural signals from the two eyes, supporting an unconventional view of the initial stages of stereopsis [28]. The study overthrew the traditional thought that stereopsis was achieved by combining signals from neurons that simultaneously detected objects in disparate parts of the two eyes’ images and verified that binocular neurons that encoding the sum and the difference between the two stereo-halves are used for stereopsis. In our previous research, we evaluated the quality of stereo images from two perspectives:the objective quality of either image of the viewpoint pair and corresponding stereo sense among viewpoints [29]. In our experiment, we found that there was a strong correlation between the difference map and correlated disparity. In other words, difference map can be treated as an alternative of binocular disparity to evaluate the stereopsis which can also be called the stereo sense quality. While the summation contains the information of both views, the measurement of the summation can be regarded as the evaluation of image quality. The combination of the image quality and stereo sense quality derives from the overall perceived quality. In this paper, based on this new study and some low-level characteristics of the HVS, a full reference objective quality assessment metric is proposed for stereo images. Main contributions of our paper are as follows: 1). Analyze and present a brief introduction of related binocular visual characteristics. 2). Propose a novel idea of separately evaluating the quality of binocular summation and binocular difference. The former can be regarded as the image quality and the latter can be deemed as the stereo sensing quality. 3). In our metric, we do not estimate a disparity map, but use difference map instead. This innovation improves the efficiency of the proposed metric greatly and provides directions for the design of stereoscopic image quality algorithm without estimating a disparity map.

The rest of this paper is organized as follows. Section II introduces the theory of binocular addition and subtraction briefly. Section III presents the detailed implementation of the proposed metric. Section IV describes the experimental results and analysis. In Section V, we summarize our conclusions.

## Binocular Summation Channel and Binocular Difference Channel

**Fig 1:**
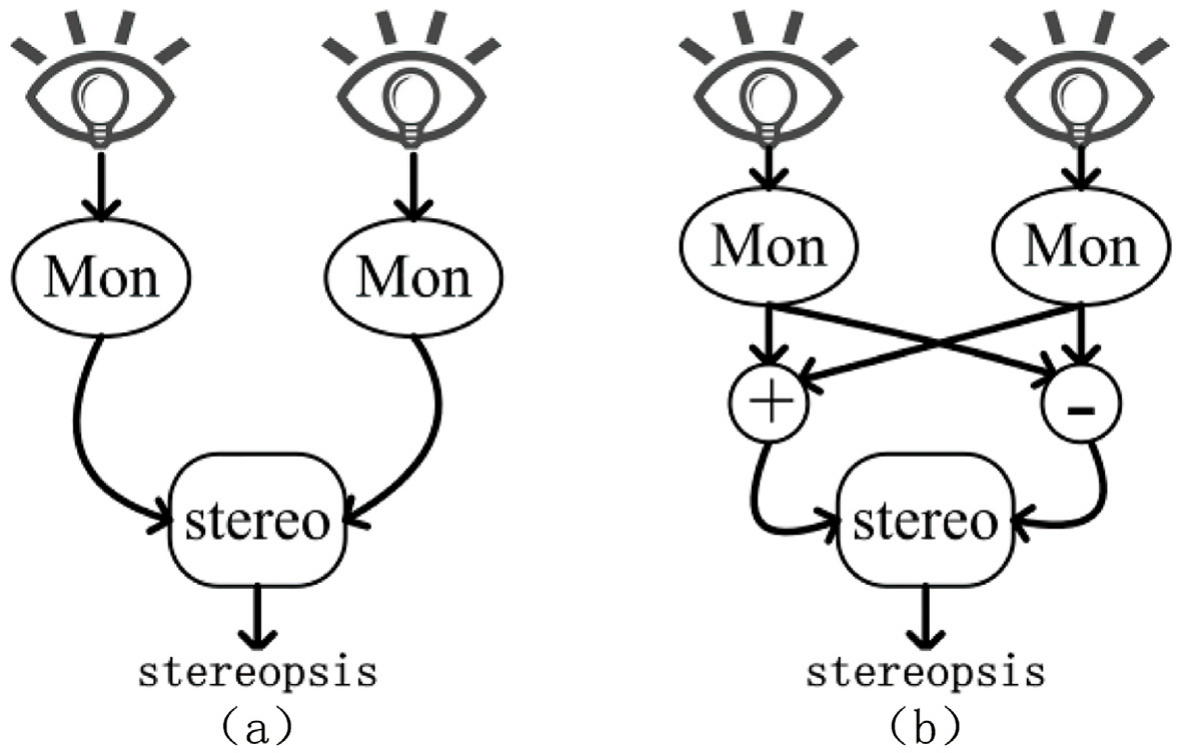
Two alternative hypothesis of the process of stereopsis, (a) Schematic diagram of traditional view, (b) Schematic diagram of recent discovery.

The human visual system (HVS) is a complicated self-adapted optical nervous system which responds to the stimulation of external light signal and forms visual sense [30]. Our eyes view the world from slightly different angle, and the resulting small differences between the images in the two eyes are the basis of the stereopsis. Traditionally, it is believed that the stereopsis was achieved by combining the signals from the neurons that simultaneously detect the objects in disparate parts of the two eyes’ images (shown in Fig 1(a)) [31]. However, an alternative view suggests that the binocular neurons first encode the summation and the difference between the two stereo halves and use them for stereopsis [32]. The speculation of the summation channel and difference channel has a long history. Until recently, an ingenious experiment designed by May *et al*. has produced convincing evidence that such channels exist [33]. The binocular summation channel (called the B+ channel) and binocular difference channel (called the B− channel) are independently adjustable (shown in Fig 1(b)). If there is no difference between the two stereo views, the binocular difference map would be blank, so the binocular difference map reveals the disparity between the two stereo views which are detected by the brain and used to construct the three-dimensional view. The binocular summation map shows the superposition of two views. With the help of binocular summation map, the system gets the ability to independently adjust the gains, or respond to strengths of the two channels which enable the system to compensate for the relatively weak B− signal found in the images of natural scenes. There are two advantages of having B+ and B− channels:

1. Because the left and right images of the stereo-pair are very similar and highly correlated, there is plentiful redundancy in the responses to the visual neurons that encode them. One way to reduce the redundancy is to convert the responses into sums and differences, as these are uncorrelated.
2. By taking the sums and differences of cone signals, the HVS produces a luminance-sensitive channel and color-sensitive channels, separately. These channels enable the HVS to ‘decouple’ the cone signals to improve the efficiency of information transmission along the visual pathway and enhance the ability to distinguish the luminance from color.

## 1 The Proposed Metric

**Fig 2:**
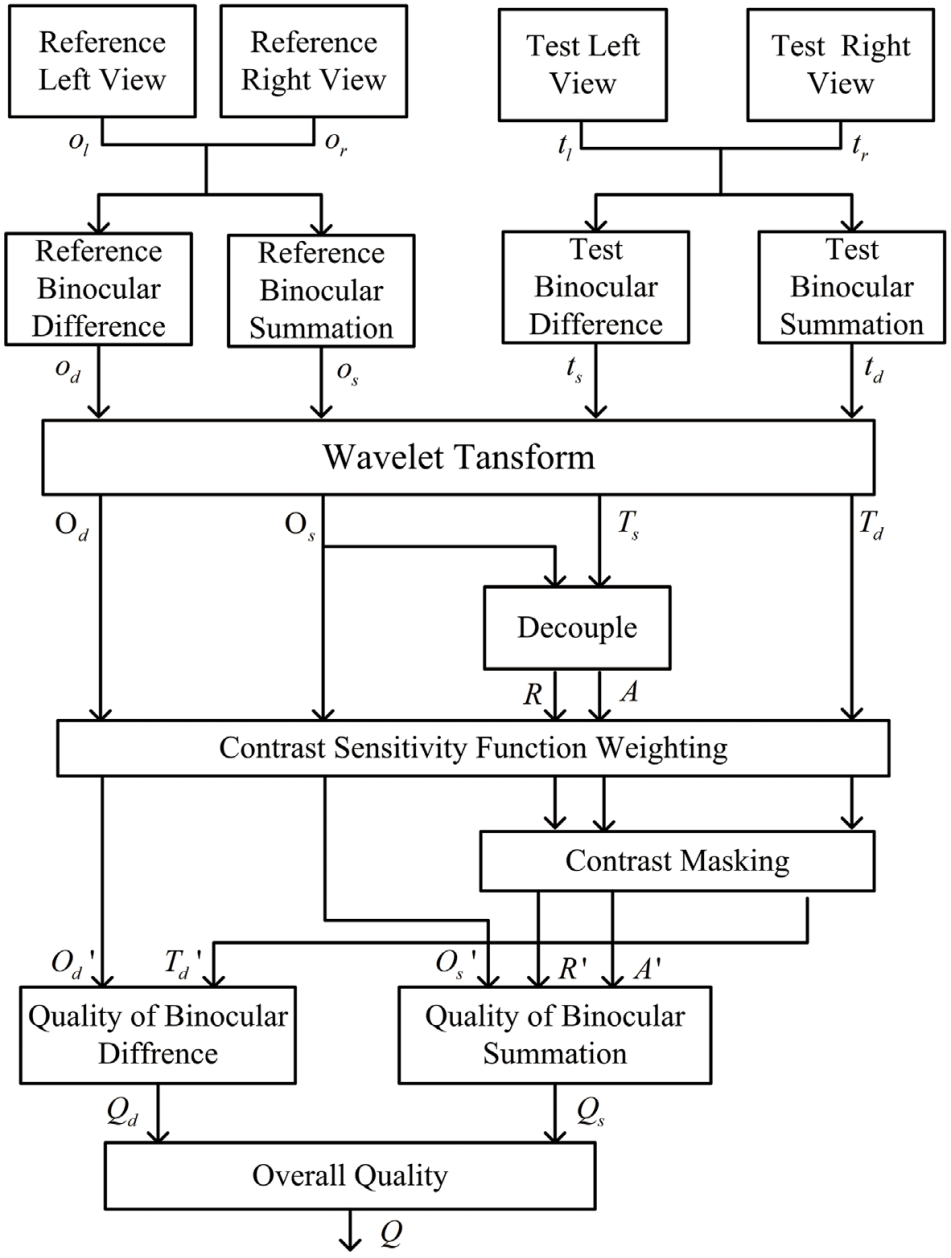
Structure of the proposed metric.

The proposed metric is a full-reference objective methodology that works with luminance only and color inputs will be converted into gray scale for further analysis. The frame work of the proposed metric is shown in Fig 2. The process of our metric can be summarized into four steps: 1. Obtain binocular summation map and binocular difference map, and analyze their characteristics. 2. Simulate the multi-channels and decouple binocular summation into two parts for further assessment. 3. Calculate the coefficient value by using the contrast sensitivity function and contrast masking. 4. Obtain the overall assessment by the nonlinear mapping and the adaptive combination of the quality of binocular summation and difference. Each section and the structure of the proposed metric are interpreted in details in the following parts.

### Binocular Summation and Binocular Difference

The experiments conducted in our previous work discovered that the absolute difference map of the viewpoint pair with human standard disparity was an approximate contour which implies that disparity performs most obviously on the edge of the objects [34]. Similar to the dense depth map, the absolute difference map also contains the high correlation information of stereo images and can be an alternative choice of the depth map to evaluate depth. The binocular summation map of test stereo pair contains the summation of both additive impairments and detail losses. Detail losses refer to the loss of useful visual information in the test stereo pair, while additive impairments refer to the redundant visual information which does not exist in the original but appears in the test pair only. By decoupling detail losses and additive impairment from the binocular summation of the test image and comparing the difference with the reference summation, we can evaluate the quality of binocular summation.

**Fig 3:**
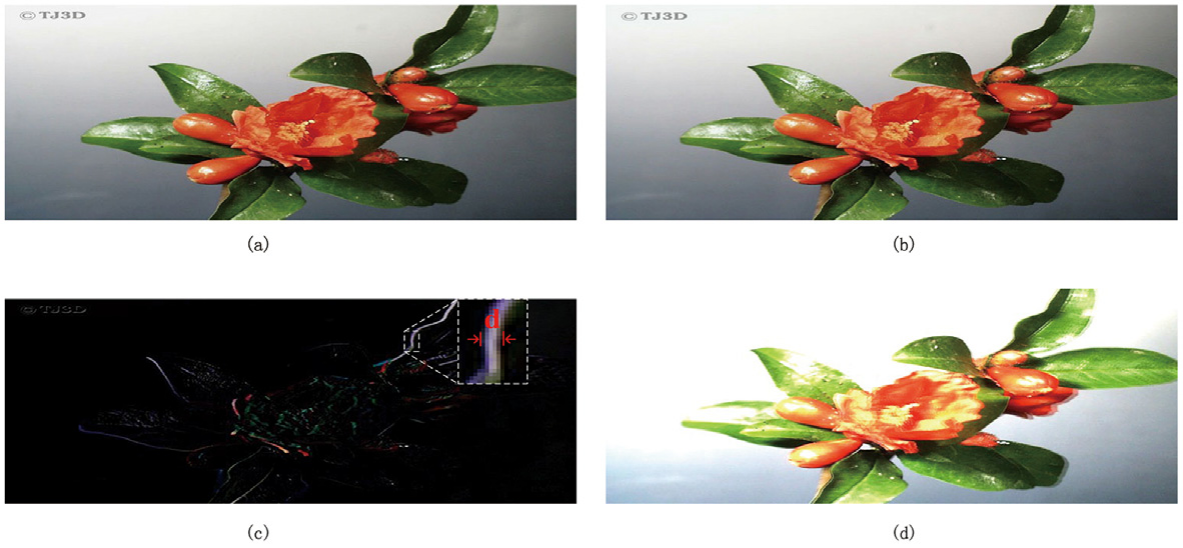
Summation and difference of stereo pair (a) Left view, (b) Right view, (c) binocular difference map and (d) binocular summation map.

Given *L* (left image) and *R* (right image), the binocular summation (*S*) map and binocular difference (*D*) map of a pair of stereo images can be calculated based on Eq (1) (shown in Fig 3).

$$\left\{\begin{array}{c} D=|L-R|\\ S=L+R \end{array}\right.$$ (1)

### Simulating Multi-channels and Decoupling Binocular Summation

**Fig 4:**
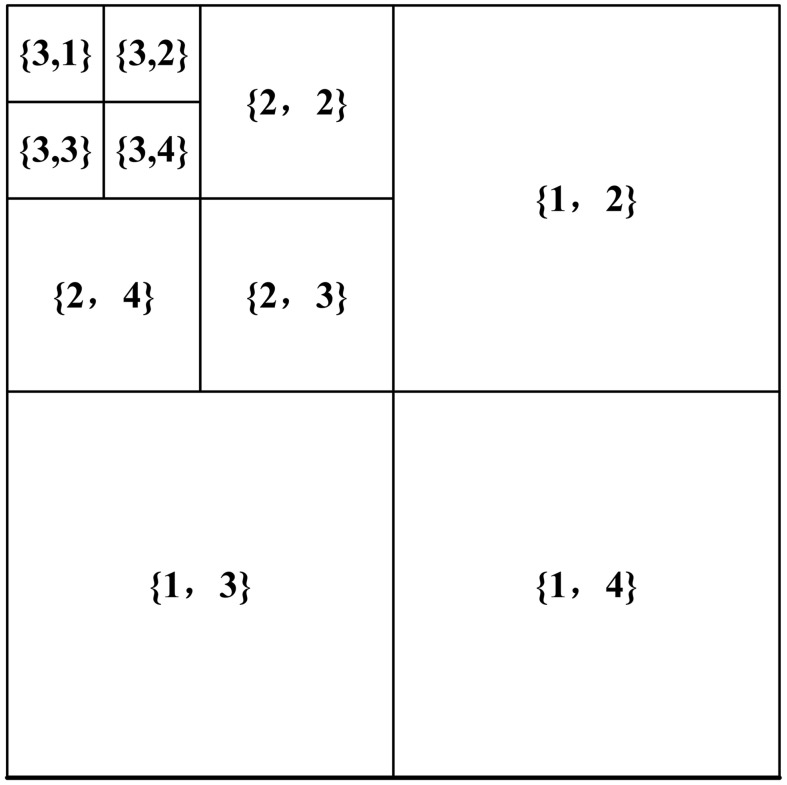
Subbands indexing of DWT. Each subband is indexed by a level λ and an orientation *θ*. *θ* = 2, 3, 4 denote the vertical, horizontal, and diagonal subbands, respectively.

Different types of neurons will be selectively sensitive to certain types of stimulation, such as the patterns of a particular frequency or orientation [35]. The visual psychology and physiology experiments have proven that there exist several independent frequency processing units in the HVS, which we call the multi-channels. Vimal and Ram found that there were several multiple frequency channels between 30° and 60° in human black-and-white vision; for human color vision, the similar channels are also found between 60° to 130° [36]. The existence of these independent channels has explained a variety of perceptual phenomena and offered a suggestion to divide visual excitation into several subbands at first for further process. To simulate this feature, we took process of discrete wavelet transform (DWT) to simulate this property (shown in Fig 4). In DWT, the inputting map is decoupled into different frequency subbands along with three different orientations (horizontal, vertical and diagonal).

**Fig 5:**
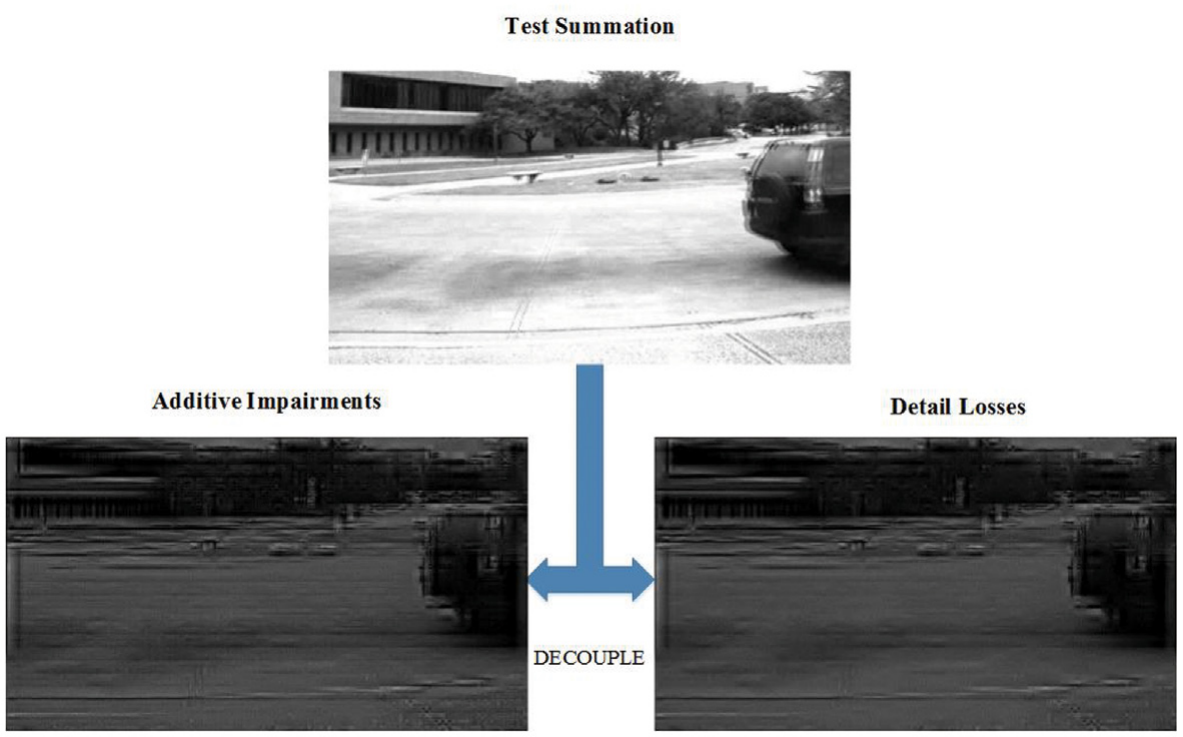
Decoupling Result of Test Binocular Summation.

As discussed in the previous section, the binocular summation of the test stereo pair contains the summation of both additive impairments and detail losses of two views. However, how to decouple the detail losses from the additive impairments was a key problem we were faced with. The algorithm proposed by Li makes it possible for us [37]. By using this algorithm, a test image is decoupled into two parts: the restored image (exhibits the same quantity of detail losses as the test image but is additive impairment free) and the additive impairments (contain no original image content but additive noises only) (shown in Fig 5). There are two advantages of using this decoupling algorithm:

1. Unlike some other existing restoration algorithms used for de-noising, de-blocking, de-blurring, super-resolution, etc., which intend to recover the original image as perfectly as possible by compensating the detail losses, the decoupling algorithm exhibits the same number of detail losses as the test image.
2. The decoupling algorithm is easy to implement with a small number of calculations.

By separately evaluating the quality of restored image and additive impairment and combining them in a proper way, we can easily gain the quality of binocular summation.

The DWT coefficients of original summation *O*_*s*_(λ, *θ*, *i*, *j*) and test summation *T*_*s*_(λ, *θ*, *i*, *j*) are given, where ((λ, *θ*, *i*, *j*)) is the index of DWT subbands, λ is the level of the conversion, *θ* = 1 indicates the approximate subband, *θ* = 2, 3, 4 denotes the horizontal, vertical and diagonal subbands, respectively. We can decouple *T*_*s*_(λ, *θ*, *i*, *j*) as according to Eq (2).

$$R\left(\lambda ,\theta ,i,j\right)=\left\{\begin{array}{cc} T_{s}\left(\lambda ,\theta ,i,j\right), & \theta =1\\ k\left(\lambda ,\theta ,i,j\right)\cdot O_{s}\left(\lambda ,\theta ,i,j\right), & otherwise \end{array}\right.$$ (2)

Where, *R*(λ, *θ*, *i*, *j*) indicates the DWT coefficients of restored images which contain detail losses of the test summation, *k*(λ, *θ*, *i*, *j*) indicates the scaled factor of each DWT coefficient of test summation and is constrained as: *k*(λ, *θ*, *i*, *j*) ∈ [0, 1]. *k*(λ, *θ*, *i*, *j*) is determined by the Eq (3).

$$k\left(\lambda ,\theta ,i,j\right)=min\left(max\left(\frac{T_{s}\left(\lambda ,\theta ,i,j\right)}{O_{s}\left(\lambda ,\theta ,i,j\right)+10^{-30}},0\right),1\right)$$ (3)

where the constant 10^−30^ is introduced to avoid dividing by zero. Since DWT is a linear operation, we can directly get the DWT coefficient of additive impairment from the Eq (4).

$$A\left(\lambda ,\theta ,i,j\right)=T_{s}\left(\lambda ,\theta ,i,j\right)-R\left(\lambda ,\theta ,i,j\right)$$ (4)

For some special cases like the contrast enhancement, the visual quality will be improved instead of degradation, as long as the image contrast is too high to look natural. For these cases, instead of relaxing the constraint on the value, we use Eq (5) to distinguish the contrast enhancement. Each pair of DWT coefficient of original summation (*O*_*s*_(λ, *θ* = 2, *i*, *j*) and *O*_*s*_(λ, *θ* = 3, *i*, *j*)) can be represented by using a point in the angular space.

$$\begin{array}{c} \psi_{t}\left(\lambda ,i,j\right)=\arctan\left(\frac{O_{s}\left(\lambda ,\theta =2,i,j\right)}{O_{s}\left(\lambda ,\theta =3,i,j\right)+10^{-30}}\right)\\ +\pi \times u(-O_{s}\left(\lambda ,\theta =3,i,j\right) \end{array}$$ (5)

Where *u*(•) refers to the unit step function. Similarly, the angle of test summation is obtained. Then the absolute angle difference can be gained from Eq (6).

$$\psi_{diff}\left(\lambda ,i,j\right)=\left|\psi_{t}\left(\lambda ,i,j\right)-\psi_{o}\left(\lambda ,i,j\right)\right|\cdot \frac{180}{\pi }$$ (6)

Different from other types of distortions, contrast enhancement will result in very small *ψ*_*diff*_(λ, *i*, *j*). In our experiment, the threshold of *ψ*_*diff*_(λ, *i*, *j*) is set to 1°. Therefore, Eq (2) can be replaced by Eq (7).

$$\begin{array}{c} R(\lambda ,\theta ,i,j)=\\ \left\{\begin{array}{cc} T_{s}\left(\lambda ,\theta ,i,j\right), & \theta =1\;or\;\psi_{diff}<1^{{}^{\circ}}\\ k\left(\lambda ,\theta ,i,j\right)\cdot O_{s}\left(\lambda ,\theta ,i,j\right), & otherwise \end{array}\right. \end{array}$$ (7)

### 1.1 Contrast Sensitivity Function and Contrast Masking

Contrast sensitivity refers to the minimum contrast value for a viewer to detect a stimulus. It is also the reciprocal of the contrast threshold. In psychovisual experiments of CSF, the subjects are required to view the sequential simple stimuli, like sine-wave gratings or Gabor patches [38]. These stimuli are presented with its contrast which gradually increases or decreases. The contrast is determined when the stimulus is just perceived or unperceived by the observers.

**Fig 6:**
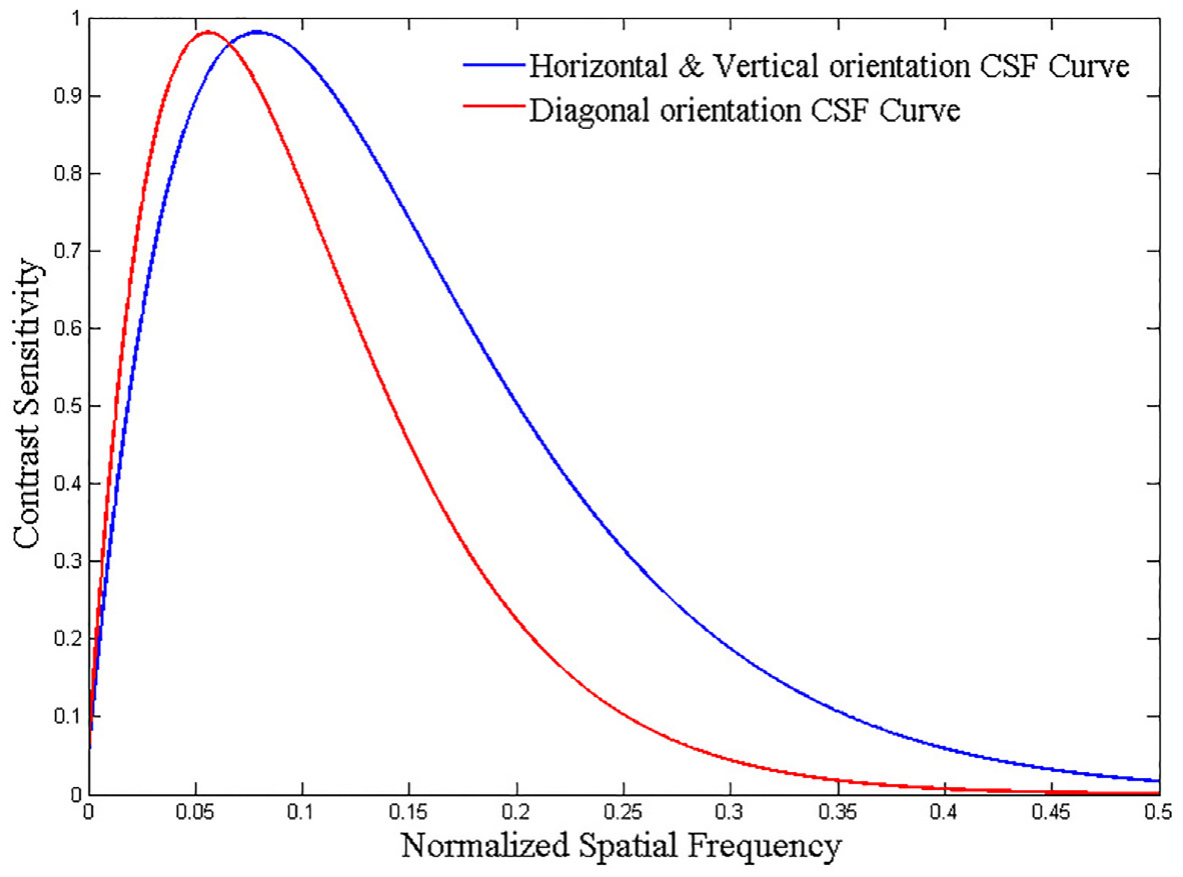
CSF curve.

The experiments indicate that the contrast sensitivity of the HVS depends on the characteristics of the stimuli such as orientation, and spatial frequency. The CSF curve peaks at the middle frequency and drops with both increasing and decreasing frequencies. Fig 6 shows the CSF curves of horizontal or vertical orientation and diagonal orientation. In our experiments, we used the CSF model proposed by Zhang [39] The CSF model can be generalized as follows:

H(ω)=(a+b·ω)·exp(-c·ω) (8)

Where, *a* = 0.31, *b* = 0.69, *c* = 0.29 and *ω* is the spatial frequency in cycles per degree (*cpd*) of visual angle. The nominal spatial frequency *F*(λ, *θ*) of each level λ can be obtained from Eq (9).

$$\begin{array}{c} \left\{\begin{array}{cc} F\left(\lambda ,\theta \right)=\frac{\pi \cdot f_{s}\cdot d}{180\cdot h\cdot 2^{\lambda }}\; & \theta =2,3\\ F\left(\lambda ,\theta \right)=\frac{\pi \cdot f_{s}\cdot d}{180\cdot h\cdot 2^{\lambda }\cdot \sqrt{2}}, & \theta =1 \end{array}\right. \end{array}$$ (9)

By calculating the contrast sensitivity function, the CSF weighed factors can be obtained and assigned to each subband.

Contrast Masking (CM) is the visibility threshold caused by the presence of a masker signal. Masking occurs when a stimulus that is visible by itself can no longer be perceived due to the presence of another stimulus [39]. In psychovisual experiment, the threshold is derived by superposing the target signal onto the masker signal. Generally, the experiment reveals that masker contrast is higher, and similarity between the target and the masker in spatial frequency is closer, and that orientation or phase leads to a higher target visibility threshold. Watson suggested that the contrast masking should be calculated over a broad range of orientation but only a limited range of space and spatial frequencies [40]. Therefore, we simply used Eq (10) to calculate the CM thresholds.

$$\begin{array}{c} MT_{\lambda }=\sum_{\theta =1}^{3}|M_{\left(\lambda ,\theta \right)}|⊗w \end{array}$$ (10)

**Fig 7:**
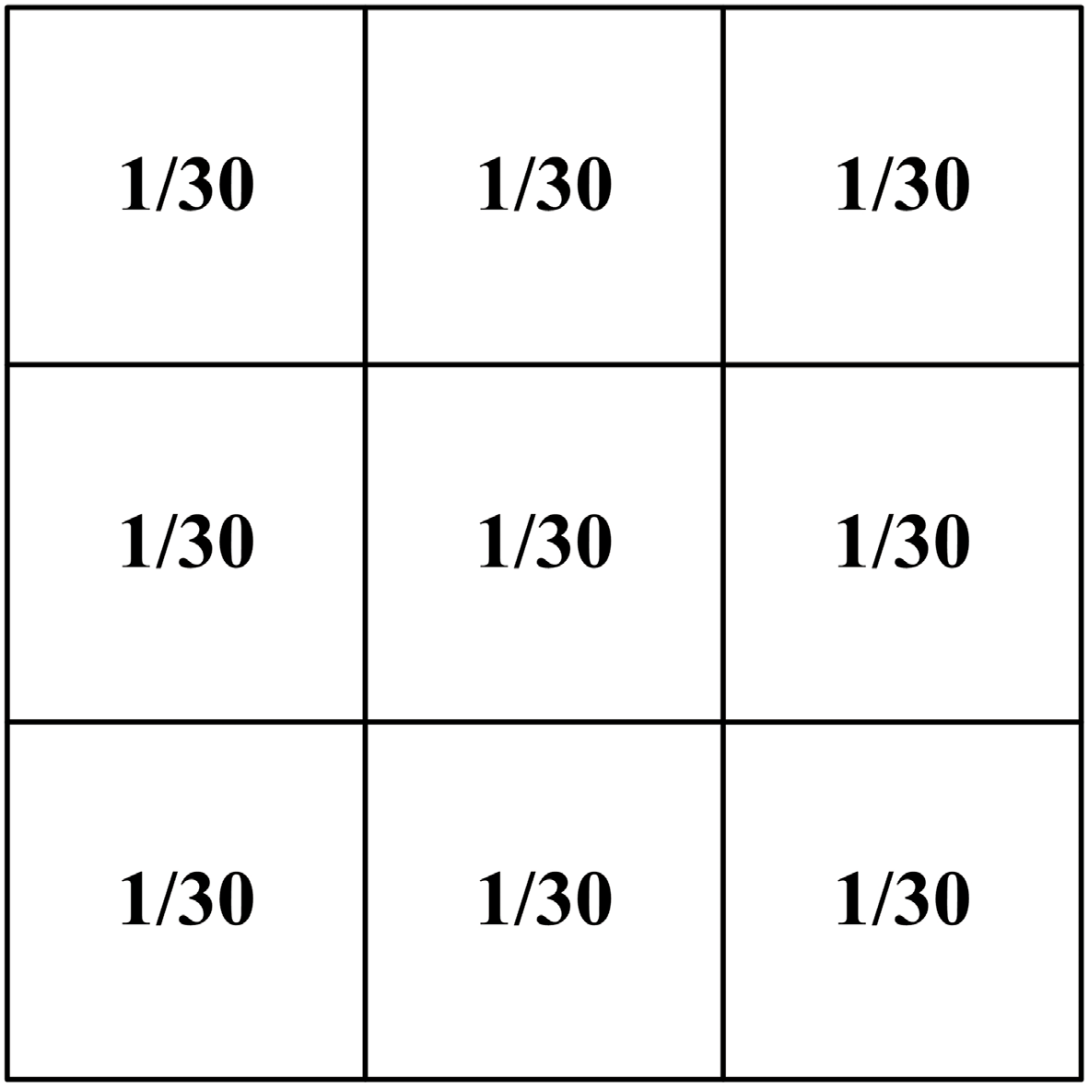
Weighting matrix *w* used in contrast masking.

Where, |*M*_(λ, *θ*)_| is the absolute DWT sub-band of masker signal, *w* is a 3 × 3 weighting matrix (shown in Fig 7) and operator ⊗ indicates convolution. For restored image and additive impairment, one’s presence will affect the visibility of the other. Therefore they are treated as the masker of each other. While for the binocular difference of the test image, the original binocular difference map can serve as the masking signal to adjust the distortions. We simply took it as the masker.

Finally, we got the result of contrast masking(*A*′, *R*′,${T_{d}}^{\prime }$) by taking the absolute CSF-weighted DWT coefficients, to subtract the corresponding CM threshold obtained from Eq (10), and clip the resultant negative values to 0. For original binocular summation and difference, we just calculated their CSF-weighted coefficients for further calculation without contrast masking processing.

### 1.2 Overall Assessment Performance

After CSF-weighting and contrast masking, we can get the masked additive impairments of binocular-summation *A*′, restored image of binocular-summation *R*′ and masked test image of binocular-difference ${T_{d}}^{\prime }$. For the assessment of binocular summation, we adopted the Minkowski merger to pool the detail loss and additive impairment. The pooling equivalent is given in Eq (11).

$$\begin{array}{c} \left\{\begin{array}{c} q_{1}=\frac{\sum_{\lambda =1}^{4}\sum_{\theta =2}^{4}{\left[\sum_{i,j\epsilon center}R^{\prime }{\left(i,j,\lambda ,\theta \right)}^{\beta_{s}}\right]}^{1/\beta_{s}}}{\sum_{\lambda =1}^{4}\sum_{\theta =2}^{4}{\left[\sum_{i,j\epsilon center}O_{s}^{\prime }{\left(i,j,\lambda ,\theta \right)}^{\beta_{s}}\right]}^{1/\beta_{s}}}\\ q_{2}=\frac{\sum_{\lambda =1}^{4}\sum_{\theta =2}^{4}{\left[\sum_{i,j\epsilon center}A^{\prime }{\left(i,j,\lambda ,\theta \right)}^{\beta_{s}}\right]}^{1/\beta_{s}}}{N} \end{array}\right. \end{array}$$ (11)

Where *N* is the number of pixels and *i*, *j* ∈ *center* means that only the central region of each subband is used in the pooling. It is used to overcome the edge effect of the wavelet transform. *β*_*s*_ is the polling factor determined. The final assessment score of binocular-summation can be gained from Eq (12) by combining *q*1 with *q*2.

$$Q_{s}=q_{1}+\alpha_{1}\cdot \left(0.5-\frac{1}{1+exp(\alpha_{2}\cdot q_{2})}\right)$$ (12)

For the assessment of binocular difference, as discussed in section *II* − *A*, the absolute disparity map is an approximate contour which contains the high correlation information of stereo images. Therefore, we adopted the CSF-weighted structural similarity metric to evaluate the quality of the binocular difference as shown in Eq (13).

$$Q_{d}=\sum_{\lambda =1}^{4}\sum_{\theta =1}^{4}\omega_{\lambda ,\theta }\cdot \frac{\left(2\mu_{x,y}+C_{1}\right)\left(2\sigma_{x,y}+C_{2}\right)}{\left(\mu_{x}^{2}+\mu_{y}^{2}+C_{1}\right)\left(\sigma_{x}^{2}+\sigma_{y}^{2}+C_{2}\right)}$$ (13)

Where, *μ*_*x*_ and *μ*_*y*_ are the average of *T*_*d*_(λ, *θ*) and *O*_*d*_(λ, *θ*), respectively. *σ*_*x*_ and *σ*_*y*_ are their variance, *σ*_*x*_ *y* is their covariance, *ω*_λ, *θ*_ is the CSF-weighted factor assigned to each orientation of each sub-band, *C*_1_ and *C*_2_ are constants determined in the experiments.

In order to combine the assessment of binocular summation with the assessment of binocular difference, a necessary step is nonlinear mapping. The nonlinear function applied in our experiment is as shown in Eq (14).

$$\begin{array}{c} Q^{\prime }=\beta_{1}\cdot \left(0.5-\frac{1}{1+exp(\beta_{2}\cdot \left(Q-\beta_{3}\right))}\right)+\beta_{4}\cdot Q+\beta_{5} \end{array}$$ (14)

After nonlinear mapping, the overall quality score can be expressed as Eq (15).

$$Q=\omega_{s}Q_{s}^{\prime }+\omega_{d}Q_{d}^{\prime }$$ (15)

Where ${Q_{s}}^{\prime }$ and ${Q_{d}}^{\prime }$ are the nonlinear mapping result of *Q*_*s*_ and *Q*_*d*_, respectively. *ω*_*s*_ and *ω*_*d*_ are constants determined by the experiment and restricted by Eq (16).

$$\begin{array}{c} \left\{\begin{array}{c} \omega_{s}+\omega_{d}=1\\ 0<\omega_{s}<1\\ 0<\omega_{d}<1 \end{array}\right. \end{array}$$ (16)

## Experimental Result and Analysis

### Stereo Database

**Fig 8:**
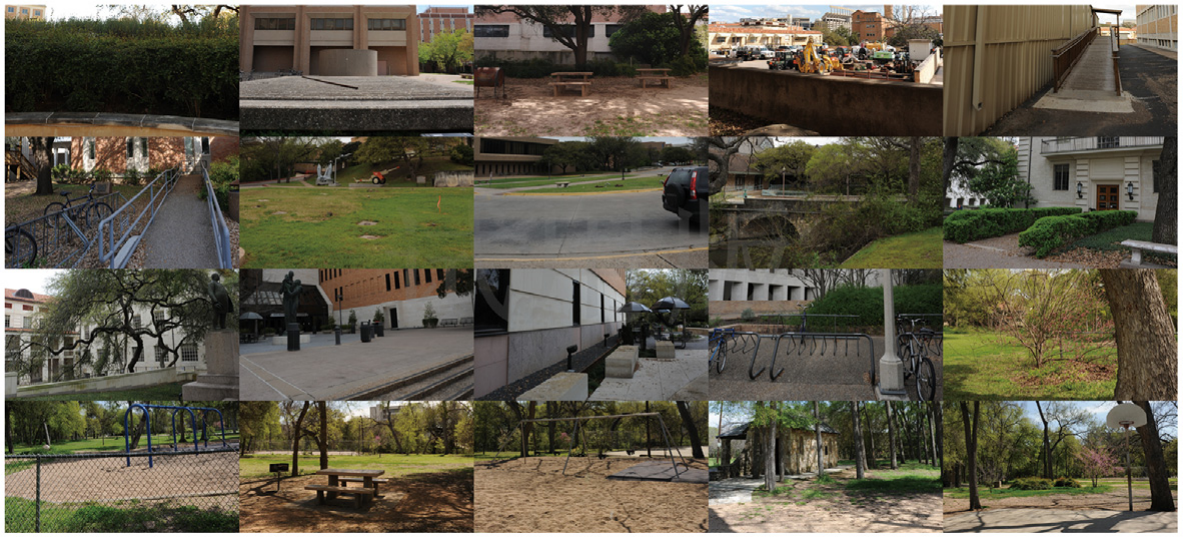
20 left views of original stereo image pairs.

**Fig 9:**
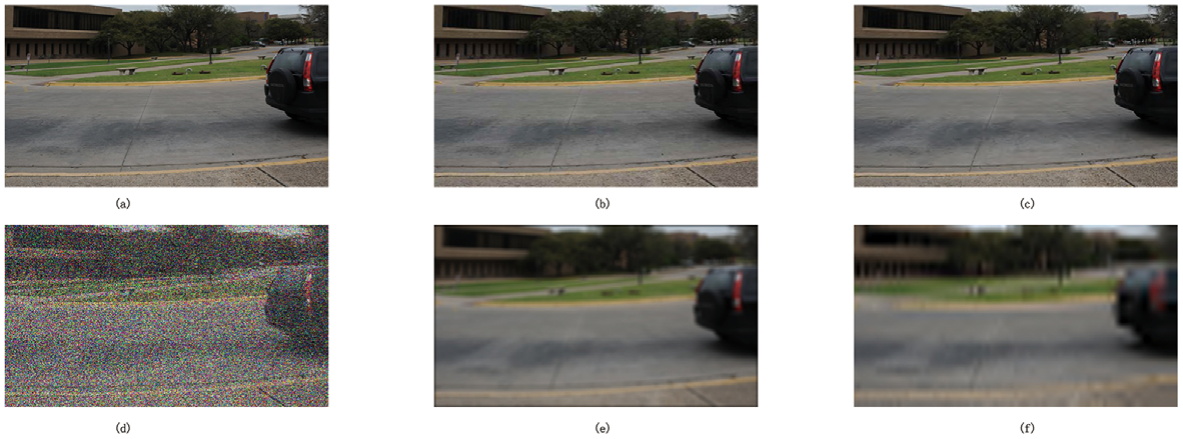
One of the original left view images and the corresponding five types of distortion. (a) Original left view. (b) JEPG compressed. (c) JEPG 2000 compressed. (d) White noise. (e) Blur. (f) Fast fading.

The stereo subjectively-rated image database used in our experiment is LIVE 3D Image Quality Database of the University of Texas at Austin (phase 1) [19]. The main interest of this database is the availability of subjective scores which allows the study on the correlation between the experimental results and human judgment. Besides, the most existing metrics for stereoscopic images work on the database of their own, which are either private or unavailable for us to compare or lack in subjective ratings. To get a more objective and impartial evaluation, we adopted the third party database. We selected 20 pairs of original stereo images (shown in Fig 8) and 365 pairs of distorted stereo images for comparison. Distorted stereo pairs contain five types of distortions, including JEPG, JEPG2000 compression, additive white noise, blur and fast fading (shown in Fig 9).

### Performance Measures and Parameters Determination

To evaluate the predictive performance of the proposed metric, four common performance measures, including the Pearson linear correlation coefficient(*PLCC*), the root mean squared error (*RMSE*), the Spearman rank-order correlation coefficient (*SROCC*) and Kendall rank-order correlation coefficient (*KROCC*), were adopted. The *PLCC* and *RMSE* are used to measure the prediction accuracy and are defined in Eqs (17) and (18). The *SROCC* and *KROCC* are used to evaluate the prediction monotonicity. For a perfect match of objective and subjective scores, *RMSE* = 0 and *PLCC* = *SROCC* = *KROCC* = 1.

$$\begin{array}{c} PLCCC\left(X,Y\right)=\frac{\sum_{i=1}^{n}\left(X_{i}-\bar{X_{i}}\right)\left(Y_{i}-\bar{Y_{i}}\right)}{\sqrt{\sum_{i=1}^{n}{\left(X_{i}-\bar{X_{i}}\right)}^{2}\sum_{i=1}^{n}{\left(Y_{i}-\bar{Y_{i}}\right)}^{2}}} \end{array}$$ (17)

$$\begin{array}{c} RMSE\left(X,Y\right)=\sqrt{\frac{1}{n}\sum_{i=1}^{n}{\left(X_{i}-Y_{i}\right)}^{2}} \end{array}$$ (18)

In the proposed metric, five parameters need to be determined i.e., the pooling component *β*_*S*_ in Eq (12), *α*_1_, *α*_2_ in Eq (13), and weighting factors *ω*_*S*_, *ω*_*d*_ in Eq (18). The strategy we used in parameterization is to select the parameters, which depends on how well the resulting models match the physiological and psychophysical experiment. The parameter result is *β*_*S*_ = 3, *α*_1_ = 1.1, *α*_2_ = 515, *ω*_*S*_ = 0.465, and *ω*_*d*_ = 0.535.

### Overall Performance

**Fig 10:**
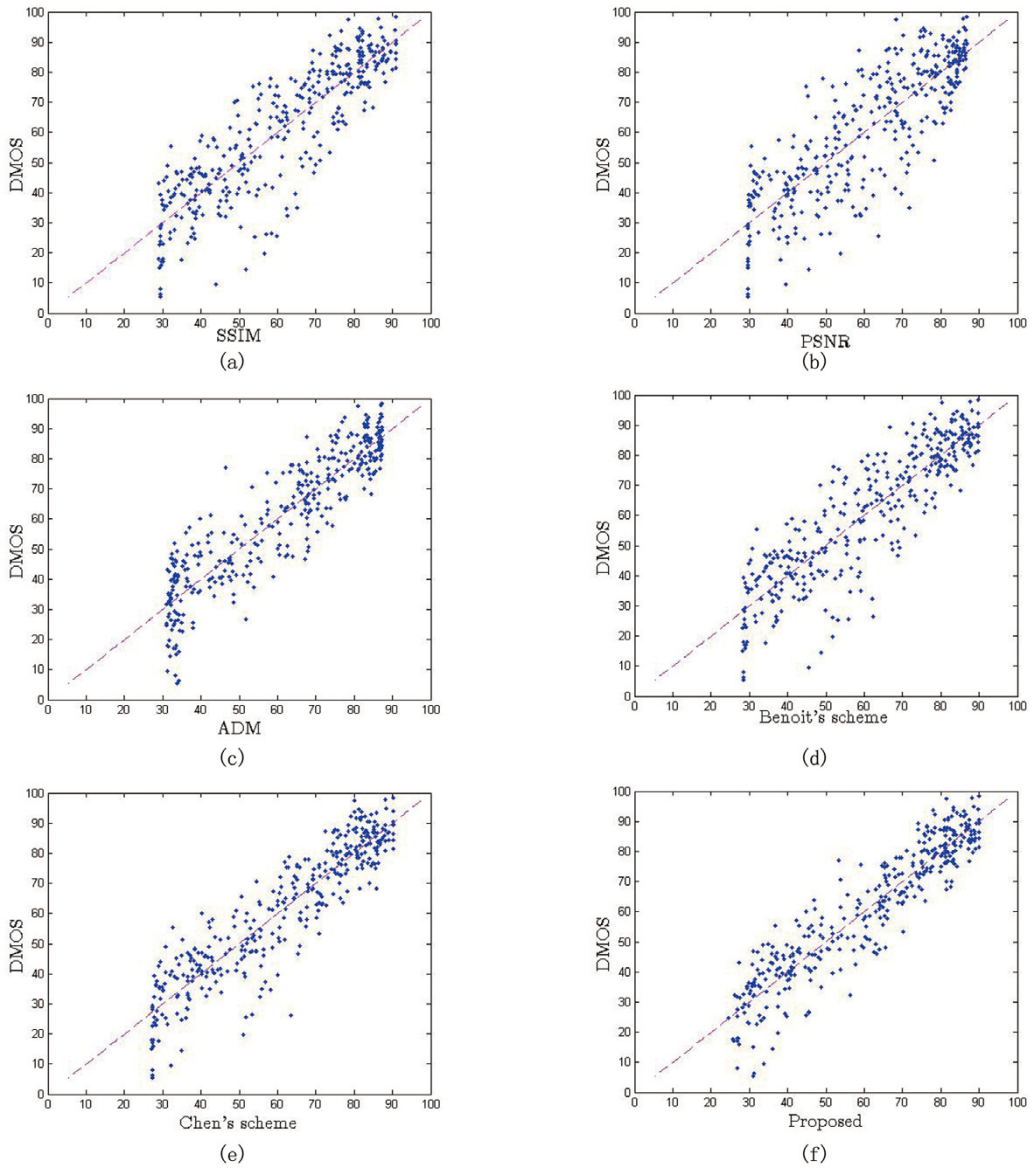
Scatter plots of objective scores versus subjective scores for the eight metric, (a)SSIM, (b)PSNR, (c)ADM, (d) Benoit’s scheme, (e) Chen’s scheme, and (f) Proposed scheme.

In this section, we compared the performance of the proposed metric with several implementations of commonly used 2D metrics i.e. PSNR, SSIM and ADM [37], and two existing schemes for stereo images, Benoit’s scheme [17], Chen’s scheme [19] and Lin’s scheme [20]. The score measured by 2D metrics will be given as the average score of the left and right view. For Benoit’s scheme, we adopted the *d*1 metric proposed in the paper, in which the 2D metric is SSIM and disparity distortion is estimated by using the global correlation coefficient between the original and distorted disparity maps. For Chen’s scheme, the 2D metric adopted is SSIM. Since all schemes require estimated disparity maps, we use the same stereo matching algorithm [41] to create the disparity maps. Fig 10 shows the scatter plots of six metrics respectively. The vertical axis denotes the subjective score of perceptual quality and the horizontal axis denotes the predicted score of each metric. From the scatter plots, we can see that the scatter points of the proposed metric are more concentrated to the diagonal than other metrics. The PSNR performs the worst compared with the other metrics, because the scatter points are chaotic and not obviously correlated with DMOS. The maximum error between PSNR and DMOS is up to 47% and the mean error is 30%. Therefore, the PSNR should not be used as a metric to evaluate the quality of stereo images. The scatter points of SSIM is more concentrated to the diagonal, but the maximum error and mean error are still high up to 43% and 26%, respectively. The maximum error and mean error of the proposed metric are 17% and 9% respectively, which are the lowest among all metrics.

To provide an intuitive instructions of each metric, Table 1 shows the values of *PLCC*, *RMSE*, *SROCC* and *KROCC* of each metric where the indicator that gives the best performance is highlighted in bold. From the table we can see that the proposed metric is more prominent than the other metrics. The decouple algorithm used in our metric is from [37] but our metric performs much better. It proves that stereo sensing quality plays an important role in predicting the quality of stereoscopic images. The performance of the Benoit’s scheme is lower than the 2D-IQA ADM but is better than its related 2D-IQA SSIM. On one hand it emphasizes the importance of stereo sensing quality in predicting the perceived quality of stereoscopic images and the addition of disparity can improve the performance to a certain extent; on the other hand, it reveals the deficiency of these metrics that the quality of the estimated disparity is highly dependent on the stereo matching algorithms. In our metric, we do not need to estimate a disparity map. Instead we use the difference map which is another form to show stereo sensing as a replacement. The use of difference maps not only reduces our risk that the performance of the metric depends on the stereo matching metric used, but also saves time of estimating a disparity map. The run time of each metric is given as Table 2 where the Total Time is the total elapsed time to predict the quality of all distorted stereoscopic images included in the stereo database, Average Time is the average time spent to predict the quality of a single pair, Self Time is the time spent of the main function excluding the time spent in its child functions. Self Time also includes overhead resulting from the process of profiling. From the table we can see that the running-speed of the proposed metric is faster than Benoit’s scheme and Chen’s scheme because both schemes need to estimate a disparity map. The running-speed of the proposed metric is lower than 2D-IQA but is not far behind.

**Table 1:** Performance comparison.

	PSNR	SSIM	ADM	Benoit’s scheme	Lin’s Scheme	Chen’s scheme	Proposed
PLCC	0.8345	0.8725	0.9119	0.8829	0.8645	0.9166	**0.9303**
SROCC	0.8341	0.8757	0.9083	0.8862	0.8559	0.9157	**0.9263**
KROCC	0.6297	0.6792	0.7187	0.6907	0.6559	0.7368	**0.7536**
RMSE	12.0480	10.6841	8.9752	10.2681	10.9898	8.7406	**8.0189**

**Table 2:** Run Time of each metric.

	PSNR	SSIM	ADM	Benoit’s scheme	Lin’s scheme	Chen’s scheme	Proposed
Total Time (s)	34.734 s	92.081 s	143.257 s	9268.470 s	9846.461	9309.223 s	207.127 s
Average Time (s)	0.095 s	0.252 s	0.463 s	25.393 s	26.976	25.505 s	0.567 s
Self Time (s)	3.272 s	3.149 s	2.7132 s	6.155 s	2.561 s	0.624 s	1.105 s

However a good performance of overall quality prediction does not necessarily mean a good performance for a certain distortion type. Table 3 shows the value of PLCC, RMSE, SROCC and KROCC of each distortion type. From the table we can see that PSNR is a useful measure only for additive white noise. Chen’s scheme performs better for distortion of blur and fast fading. Shao’s scheme is not very capable in dealing with the distortion of “noise”. The proposed scheme is less effective in predicting JEPG, JEPG2000 compressed distorted image quality but the overall performance of the proposed metric is better than the Benoit’s scheme and Chen’s scheme in general.

**Table 3:** Performance comparison of each distortion.

Distortions	Criteria	PSNR	SSIM	ADM	Benoit’s scheme	Lin’s scheme	Chen’s scheme	Proposed
JEPG	PLCC	0.2187	0.4871	0.6719	0.5579	0.2866	0.6342	**0.6777**
	SROCC	0.2183	0.4347	0.5926	0.5189	0.2996	0.5582	**0.6010**
	KROCC	0.1797	0.2818	0.4009	0.3490	0.2461	0.3718	**0.4805**
	RMSE	8.5079	7.6146	6.4578	7.2360	8.3531	6.7433	**6.4111**
JEPG2000	PLCC	0.7851	0.8650	0.9333	0.8897	0.8381	0.9164	**0.9376**
	SROCC	0.7993	0.8581	0.9008	0.8701	0.8388	0.8956	**0.9026**
	KROCC	0.5918	0.6563	0.7158	0.6696	0.6386	0.7139	**0.7259**
	RMSE	10.6954	8.6632	**6.0009**	7.8853	9.4210	6.9136	6.0037
White Noise	PLCC	0.9349	0.9384	0.9211	0.9360	0.9280	**0.9432**	0.9213
	SROCC	0.9316	0.9387	0.9236	0.9347	0.9284	**0.9481**	0.9264
	KROCC	0.7665	0.7848	0.7608	0.7753	0.7614	**0.8025**	0.7620
	RMSE	7.8700	7.6623	8.6342	7.8058	8.2618	**7.3690**	8.6231
Blur	PLCC	0.9156	0.9607	0.8987	0.9256	0.9475	0.416	**0.9511**
	SROCC	0.9020	0.8793	0.8891	0.8967	0.9345	**0.9261**	0.9217
	KROCC	0.7333	0.7192	0.7859	0.7354	0.7859	**0.7717**	0.7576
	RMSE	7.7594	7.6206	5.3549	7.3052	6.1721	6.4977	**5.9619**
Fast Fading	PLCC	0.7026	0.7207	**0.8549**	0.7514	0.7086	0.7575	0.8272
	SROCC	0.5875	0.5871	**0.8154**	0.6142	0.6581	0.6879	0.7734
	KROCC	0.4168	0.4308	**0.6289**	0.4561	0.4719	0.5118	0.5865
	RMSE	11.7900	11.4857	**8.5957**	10.9314	11.6895	10.8152	9.3087

**Fig 11:**
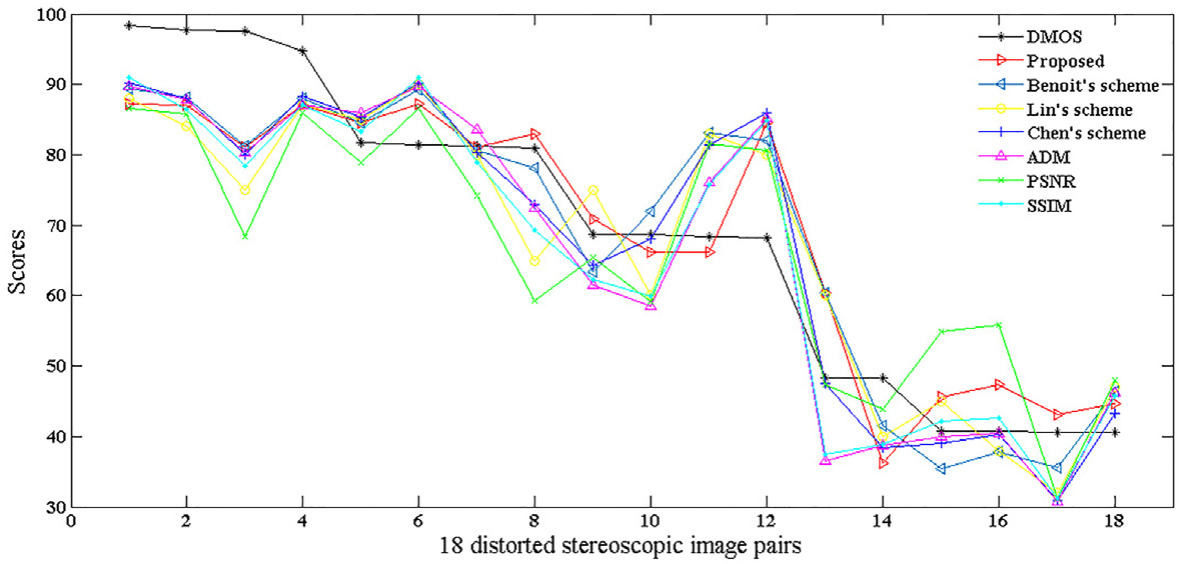
10 statistic results of 18 distorted pairs of an original pair.

To better illustrate the relationship between the DMOS and objective metrics discussed in this paper, the statistic results of 18 distorted image pairs of a stereo pair are shown in Fig 11. From the statistic results, we can see that the trend of the proposed metric is the closest to the trend of DMOS among all metrics. The Lin’ scheme, Benoit’s scheme, Chen’s scheme and ADM also show high correlation with DMOS. There’s no obvious relationship between the trend of PSNR and DMOS.

**Fig 12:**
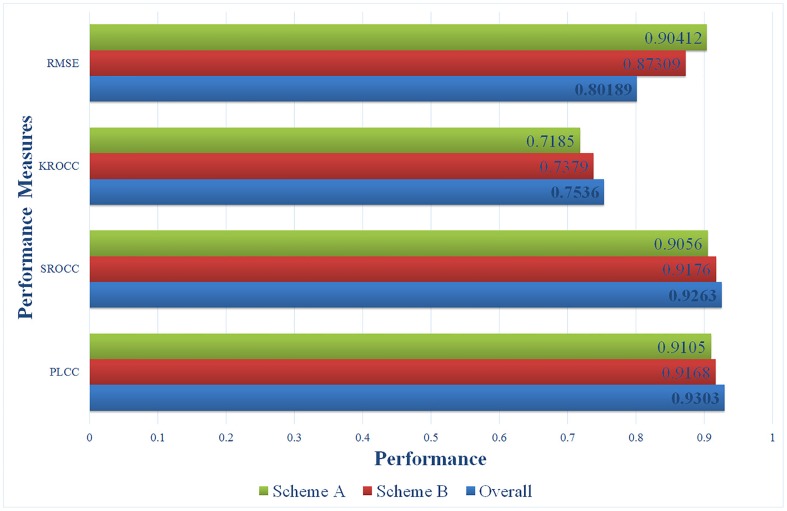
Performance comparison of each part and overall.

Since the proposed metric contains two parts, the performance of each part is also evaluated. Fig 12 shows performance of each part and the overall quality, the binocular summation part is named Scheme A while the binocular difference part is named Scheme B. In order to illustrate clearer, the RMSE value is reduced tenfold. It is obvious that the overall quality yield a higher correlation with the subjective value than both part. We further investigated the role that binocular summation and binocular difference played that contribute to this result and found that for JEPG, JEPG 2000 compression and additive white noise distortions, the score of binocular summation is high, while the score of binocular difference is relatively low. The reason is that these types of distortions make greater distortions for binocular summation but small structure distortion for binocular difference which is opposite to blur and fast fading. The overall score absorbs the advantages of binocular summation and binocular difference, which makes the overall performance consistent with subjective scores. This result however in some ways have prove the existence of binocular decouple channels of binocular difference and binocular summation at the initial phase of stereopsis.

### Result On Other Database

To further verify the proposed method, more 3D IQA Database need to be used. However, some of 3D IQA Databases are either not publicly available or absent of subjective assessment value. To solve this problem, the SVBL 3D IQA Database (Stereo Image Database of Stereo Vision and Bio-Optics Laboratory from School of Electronic and Information Engineering, Tianjin University [29]) was used to further verify the proposed method. The SVBL 3D IQA Database contains 360 distorted stereopairs and 30 original stereopairs. Each stereoscopic image in this database has bee evaluated by human subjects, and assigned a quantitative subjective quality score [42]. The performance numbers (*PLCC*, *SROCC*, *KROCC* and *RMSE*) of 2D metrics and compared 3D QA models on SVBL 3D IQA database is given in Table 4.

**Table 4:** Performance comparison on SVBL 3D database.

	PSNR	SSIM	ADM	Benoit’s scheme	Lin’s scheme	Chen’s scheme	Proposed
PLCC	0.8257	0.9060	0.8789	0.8583	0.9097	0.9143	**0.9227**
SROCC	0.0.8076	0.8986	0.8812	0.8336	0.9009	0.9103	**0.9149**
KROCC	0.5996	0.7476	0.6900	0.6349	0.7594	0.7728	**0.7392**
RMSE	12.6772	9.9756	10.6487	12.2205	10.0128	9.0385	**8.5423**

From the table we can conclude that the proposed metric performs best among all metrics. Both the proposed metric and Chen’s scheme perform better than 2D IQA metrics. Chen’s scheme performs well because it also consider some characteristics of human visual system. However the Chen’s scheme only considered binocular combination characteristic and failed in investigating the stereopsis produce process. Though Lin’s scheme considered binocular integration behaviors, its performance is mediocre. That is because on the one hand, the algorithm was specifically designed to predict the quality of 3D image compression and could not be applicable to other types of distortions, on the other hand it ignored the procedure of stereopsis. Benoit’s Scheme failed in both database, from which we can conclude that it doesn’t work to derive a stereo quality assessment metric by simply combine existing 2D metric with disparity map without considering stereoscopic visual characteristics.

## Conclusion

According to the human eye’s two processing channels: binocular summation channel and binocular difference channel, this paper proposes an objective quality assessment metric for stereoscopic images. The proposed metric introduces the binocular visual characteristics which can greatly improve the performance of the metric. Also, the proposed metric is free of depth map which makes our metric more efficient and effective. The experiment results based on LIVE 3D database and SVBL database indicate the effectiveness of the proposed quality metric in matching subjective ratings. The prosed metric have indirectly proved the existence of binocular decouple channel which are binocular summation channel and binocular difference channel which also inspire us to prove hypothesis by reasonable application. Our future work will explore application of some other HVS characteristics in quality assessment of stereoscopic images. Verify some hypothesis of stereoscopic visual characteristics by some experiments or applying the into our metric and extend our metric to stereoscopic video quality assessment.
